# Candidate Biomarkers for Genetic and Clinicopathological Diagnosis of Endometrial Cancer

**DOI:** 10.3390/ijms140612123

**Published:** 2013-06-06

**Authors:** Kouji Banno, Yuya Nogami, Iori Kisu, Megumi Yanokura, Kiyoko Umene, Kenta Masuda, Yusuke Kobayashi, Wataru Yamagami, Nobuyuki Susumu, Daisuke Aoki

**Affiliations:** Department of Obstetrics and Gynecology, School of Medicine, Keio University, Shinanomachi 35 Shinjuku-ku, Tokyo 160-8582, Japan; E-Mails: yuya22wing@hotmail.com (Y.N.); iori71march@hotmail.co.jp (I.K.); meguyano@z3.keio.jp (M.Y.); umekiyo@hotmail.co.jp (K.U.); ma-su-ken@a2.keio.jp (K.M.); kobax@a2.keio.jp (Y.K.); y_gami@msc.biglobe.ne.jp (W.Y.); susumu35@sc.itc.keio.ac.jp (N.S.); aoki@z7.keio.jp (D.A.)

**Keywords:** endometrial cancer, microsatellite instability, MSH6, DNA hypermethylation, CHFR, microRNA, Lynch syndrome, PTEN, K-ras, CA125

## Abstract

The recent increase in the frequency of endometrial cancer has emphasized the need for accurate diagnosis and improved treatment. The current diagnosis is still based on conventional pathological indicators, such as clinical stage, tumor differentiation, invasion depth and vascular invasion. However, the genetic mechanisms underlying endometrial cancer have gradually been determined, due to developments in molecular biology, leading to the possibility of new methods of diagnosis and treatment planning. New candidate biomarkers for endometrial cancer include those for molecular epigenetic mutations, such as microRNAs. These biomarkers may permit earlier detection of endometrial cancer and prediction of outcomes and are likely to contribute to future personalized therapy for endometrial cancer.

## 1. Introduction

Endometrial cancer accounts for 70% to 80% of cases of primary malignant disease in the uterus in the United States [1]. In contrast, cervical cancer accounts for most malignant uterine disease in Asia, but cases of endometrial cancer have increased, due to improved screening and Westernized dietary habits. Thus, the Japan Society of Obstetrics and Gynecology (JSOG) reported that endometrial cancer had increased from 976 patients in 1983 to 4267 in 2005 and 6113 in 2009. This most recent number accounts for about half of all cases of malignant uterine disease [2,3] and emphasizes the need to improve diagnosis and treatment of endometrial cancer.

Recent advances in molecular biology have made major contributions to the understanding of malignant diseases and development of diagnostic technology. Mutation of the *epidermal growth factor receptor* (*EGFR*) gene is an important biomarker for prediction of the effect of gefitinib, a molecular-targeted drug. Personalized medicine based on individual differences among patients is attainable using a treatment strategy with anticancer drugs chosen based on prediction of effects and adverse reactions using these biomarkers. Genetic studies of endometrial cancer have established relationships between carcinogenesis and gene mutations. Thus, mutation of *phosphatase and tensin homolog deleted from chromosome10* (*PTEN*), a tumor suppressor gene, is frequently detected in endometrioid adenocarcinoma and that of *p53* is often detected in cancer in other tissue types.

Abnormalities that cannot be explained by gene mutation are also frequently found in malignant diseases, including endometrial cancer. These include epigenetic mutations, which produce abnormal gene expression without changes in the base sequence of genomic DNA. microRNAs (miRNAs), which are associated with regulation of gene expression, are also of interest, due to their involvement in mechanisms associated with onset of malignant disease. Familial aggregation of endometrial cancer is also a focus, and patients with Lynch syndrome (hereditary non-polyposis colorectal cancer: HNPCC) are often complicated with both colorectal and hereditary endometrial cancer. Lynch syndrome is caused by germ cell mutation of mismatch repair (MMR) genes that are strongly related to endometrial cancer. These findings are likely to promote development of new treatment for endometrial cancer.

## 2. Genetic Abnormalities in Endometrial Cancer

Endometrial cancer is divided into two types [4–6]. The type 1 form occurs in patients with typical risk factors, including endocrine disorders, such as obesity and excessive estrogen, and accounts for about 80% of cases of endometrial cancer. The type 1 cancer is well-differentiated endometrioid adenocarcinoma with relatively good outcomes and frequently occurs in perimenopausal women. The type 2 form occurs in patients without the above risk factors and includes poorly differentiated endometrioid adenocarcinoma, serous adenocarcinoma, mucinous adenocarcinoma and clear cell adenocarcinoma. This form has poor outcomes and tends to develop in elderly women.

**Figure 1:**
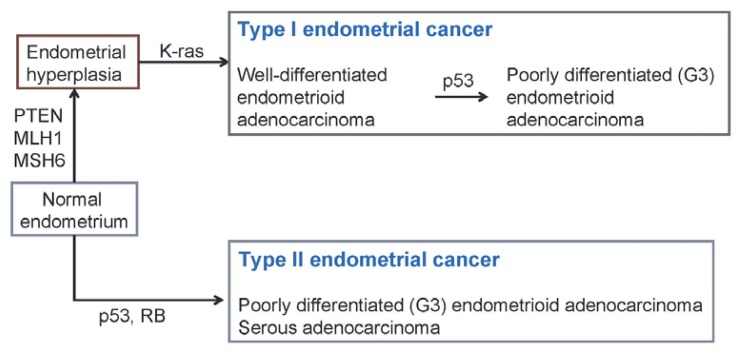
Genetic mutations in endometrial cancer.

Type 1 endometrial cancer is most commonly characterized by mutation of *PTEN*, a tumor suppressor; and also by mutations in *v-Ki-ras2 Kirsten rat sarcoma viral oncogene homolog* (*K-ras*), *β-catenin*, *phosphatidylinositol-4*,*5-bisphosphate 3-kinase*, *catalytic subunit alpha* (*PIK3CA*) and *phosphatidylinositol 3-kinase* (*PIK3*). Some cases also have inactivation of *mutS homolog 6* (*MSH6*), which is associated with microsatellite instability (MSI) [7,8]. Inherited or somatically acquired mutations of MSH6, although relatively uncommon in endometrial cancers in general, are often seen in MSI endometrial cancer. Type 2 endometrial cancer shows mutations of *p53* and *p16*, reduced expression of *E-cadherin* and overexpression of *human epidermal growth factor receptor 2* (*Her-2/neu*) (Table 1 and Figure 1) [7,9].

## 3. Epigenetic Aberrant Methylation in Endometrial Cancer

**Figure 2:**
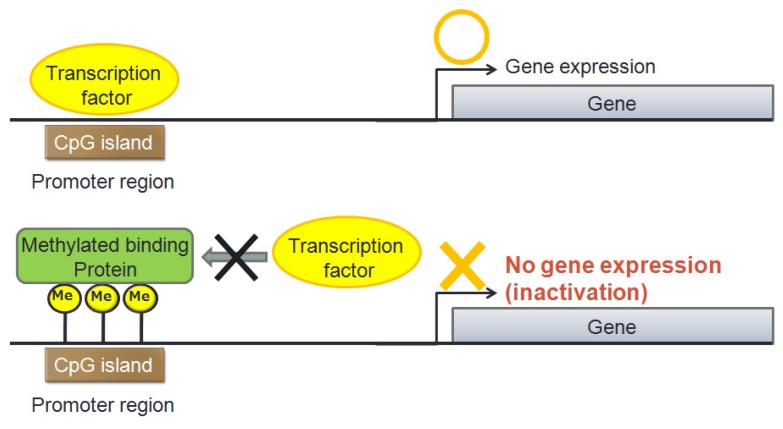
Mechanism of gene inactivation by aberrant methylation.

Epigenetic mutations cause abnormal gene expression without changes in the DNA base sequence. Epigenetic mechanisms regulate downstream gene expression through aberrant hypermethylation of CpG islands in promoter regions, hypomethylation of entire genomes and histone acetylation [10]. Aberrant hypermethylation of gene promoters causes transcriptional silencing of oncogenes, resulting in abnormal proliferation of cells and carcinogenesis (Figure 2).

The nucleosome, a unit of chromatin, consists of a core tetramer of histone pairs of H3-H4 dimers that form an octamer with 2 histone H2A-H2B dimers, with approximately 147 bp of DNA wrapping around the octamer about 1.75-times. Structural analysis of the nucleosome shows that the *N*-termini of histones protrude to sites outside the DNA. The *N* (and *C*)-terminal domains of the histone core proteins (histone tails) undergo various posttranslational modifications, including acetylation, methylation, ubiquitination and phosphorylation. Combinations of histone modifications define the status of the activation of the chromosome and positional information, and therefore, this is considered to be a kind of genetic code. Histone acetylation removes positive charges of lysine residues and decreases electrostatic interactions with the negatively charged DNA. This reduces the affinity of the histone for the DNA and leads to conversion of heterochromatin to euchromatin, allowing cofactors to interact with DNA and increase transcriptional activity.

Histone acetylation and deacetylation are catalyzed by histone acetyl transferase (HAT) and histone deacetylase (HDAC) enzymes, respectively. Histone acetylation activates binding of transcription factors and enhances transcriptional activation, while deacetylation inhibits transcription. The 18 known HDACs are classified into Classes I to IV based on amino acid sequence homology at sites of enzyme activity [11]. Class I enzymes (HDAC 1, 2, 3 and 8) are homologs of yeast Rpd3 that are present mainly in nuclei and are constitutively expressed in many cells and tissues. Class II enzymes (HDAC 4, 5, 6, 7, 9 and 10) are homologs of yeast Hda1 and are mainly present in the brain, myocardium and skeletal muscles. Class II is further classified into Classes IIa (HDAC 4, 5, 7 and 9) and IIb (HDAC 6 and 10). Class III enzymes are Sir2 homologs and include SIRT 1, 2, 3, 4, 5, 6 and 7. Class IV currently includes HDAC11 alone. The relationship between HDAC expression and endometrial malignant transformation remains unclear. Class I HDAC expression in the endometrium and in endometrial cancer has recently been shown for the first time [12,13]. HDAC expression occurs throughout the menstrual cycle, but HDAC3 expression decreases from menstrual days six to 10, suggesting that HDAC expression is involved in endometrial differentiation [12]. Endometrial cancer with strong Class I HDAC expression may have a poor prognosis [13], and it is of note that many studies have shown antitumor effects of HDAC inhibitors, including in endometrial cancer.

MSI is a phenomenon in which microsatellite repeat sequences in tumor tissues differ from those in non-tumor tissues, due to dysfunction of the repair of base sequence errors in DNA replication. MSI occurs in Lynch syndrome, is commonly used in screening and has been detected in 20% of patients with sporadic endometrial cancer [14,15]. Inactivation of *MLH1*, a DNA MMR gene, is common in endometrial cancer, due to hypermethylation in CpG islands of gene promoters, and is a major cause of MSI. In a study of 93 patients who underwent radiotherapy after total extrafascial hysterectomy, Bilbao *et al*. [16] found 20 MSI-positive cases (22%) and a relationship of MSI with progression (*p* = 0.04) and vascular invasion (*p* = 0.009). In addition, the 10-year disease-free survival (DFS) of 53.8% in MSI-positive patients was significantly lower than that of 75.9% in MSI-negative patients (*p* = 0.003) [16]. This shows that MSI may be a useful diagnostic and prognostic indicator in detection and treatment of endometrial cancer.

Aberrant hypermethylation is detected at the precancerous lesion stage [17] and is significantly more widespread in malignant lesions compared to benign tumors. In addition to *MLH1*, genes, such as *estrogen receptor α* (*ERα*), *progesterone receptor* (*PR*), *cyclin-dependent kinase inhibitor2A/P16* (*CDKN2A/P16*), *secreted frizzled-related protein1* (*SFRP1*), *SFRP2* and *SFRP5*, may be hypermethylated in malignant lesions [18], and this may allow earlier identification of these lesions.

Aberrant DNA hypermethylation of mitotic checkpoint genes has a major influence on the response to anticancer drugs. Checkpoint with forkhead-associated and ring finger (CHFR), a mitotic phase checkpoint gene, delays chromatin aggregation and progression to mitosis. CHFR acts by preventing cells from entering mitosis in response to a range of mitotic stress agents in the so-called antephase check point [19,20]. Taxanes are anticancer drugs that act in mitotic cells as microtubule depolymerization inhibitors, but do not affect cells with normal CHFR, because DNA is repaired in the G2 phase. However, in cells with CHFR inactivated by aberrant methylation, DNA damage induced by taxanes is not repaired, resulting in cell death. Therefore, these cells are highly sensitive to taxanes, and the anticancer effect of taxanes can be predicted by examining aberrant methylation of CHFR.

Banno *et al*. [21] detected aberrant methylation of CHFR in six (12%) of 50 patients with endometrial cancer, but not in nine healthy women or 10 patients with atypical endometrial hyperplasia. Aberrant methylation of CHFR was more frequent in poorly-differentiated (G3) endometrioid adenocarcinoma compared with the well-differentiated (G1) type. Among six cell lines (HHUA, SNG-II, HEC-1-B, HEC-108, HOOUA and KLE), aberrant methylation of CHFR was detected in SNG-II and HEC-108 cells, both of which are highly sensitive to paclitaxel and docetaxel. Following treatment with 5-aza-2′-deoxycytidine (5-aza-dC) for demethylation, the sensitivity of these two cell lines to paclitaxel and docetaxel was significantly decreased. These results suggest that aberrant methylation of CHFR is a biomarker of carcinogenesis and poorly-differentiated cancer and may be useful as an indicator for sensitivity to taxanes. This offers the potential for individualized therapy based on analysis of CHFR methylation.

## 4. microRNA Abnormalities in Endometrial Cancer

miRNAs are short non-coding RNAs of 20 to 23 base pairs [22] that regulate gene expression at the post-transcriptional level [23]. miRNAs are first generated as primary miRNAs (pri-miRNAs) with 100 to several thousand base pairs. Pre-miRNAs with about seven base pairs and a hairpin loop are spliced out from pri-miRNA by protein complexes, such as Drosha and DGCR8. These pre-miRNAs are transported out of the nucleus and formed into double-stranded miRNAs of about 22 base pairs by the Dicer enzyme, which has RNase activity. The double-stranded miRNAs bind to the Argonaute protein, leading to formation of the miRNA-containing RNA-induced silencing complex (miRISC). In ribosomes, miRNAs in miRISC bind to the 3′ UTR of the mRNA of a target gene and inhibit expression and translation or degrade the targeted mRNA [22].

miRNAs play an important role in carcinogenesis by targeting tumor suppressor oncogenes or by functioning as oncogenes with elevated expression [23,24]. In endometrial cancer and, particularly, in endometrioid adenocarcinoma, various miRNAs are down- or up-regulated [25–27]. The miR-200 family (miR-141, miR-200a, miR-200b, miR-200c and miR-429) [27] and miR-203 [28], miR-205 [26] and miR-210 [29] are all upregulated, whereas miR-410 [28], miR-15b, miR-17-5p, miR-20a, miR-125a, miR-214, miR-221, miR-222 and miR-424 are all downregulated [29] (Table 2). Torres *et al*. [28] showed that patients with recurrence can be differentiated by the levels of miR-205 and miR-200a with high significance (area under curve (AUC): 0.854, 95% confidence interval (CI): 0.691–0.951) and that lymph node metastasis is associated with expression of miR-200a, miR-203 and miR-429 at 80% sensitivity and 79% specificity.

Aberrant DNA hypermethylation also inactivates expression of miRNAs. For example, the gene for miR-124 is present in or around CpG islands in the chromosomal regions of 8p23.1, 8q12.3 and 20q13.33, and expression of miR-124 is inhibited by aberrant DNA methylation in colorectal cancer. In HCT116 colorectal cancer cells, methyl-CpG binding protein (MeCP2) and methyl-CpG binding domain protein (MBD2) bind to promoters of the miR-124 gene, but this does not occur in demethylated HCT116 cells [22]. Hiroki *et al*. [30] showed that 113 and 54 miRNAs were downregulated in endometrioid and serous adenocarcinoma, respectively, with six downregulated in serous adenocarcinoma only. In particular, miR-34b was markedly downregulated, due to aberrant hypermethylation. Tsuruta *et al*. [31] found normal miR-152 expression in six normal endometrium specimens, but suppression to <50% in 13 endometrial cancer cell lines. This suppression was related to aberrant DNA methylation in all cell lines and expression was frequently recovered by demethylation using 10 μmol/L 5-aza-dC. The recovery of miR-152 expression significantly decreased cell proliferation in endometrial cancer cells, which strongly suggests a tumor suppressor function of miR-152.

Endometrial cancer generally has good outcomes with surgical treatment if diagnosed at an early stage, but effective biomarkers have not been established to date. The studies described above and ongoing work suggest that miRNAs may serve as biomarkers for diagnosis and monitoring of treatment outcomes in endometrial cancer.

## 5. Diagnosis of Endometrial Cancer as a Familial Tumor

Cases of multiple occurrence of endometrial cancer in a single family suggest involvement of genetic abnormalities. The most typical are cases of Lynch syndrome, an autosomal dominant disorder characterized by juvenile-onset of malignant tumors and colorectal cancer as the core malignant tumor [32]. Patients with Lynch syndrome have risks for gastric, small intestinal, biliary and urologic cancer, in addition to colorectal cancer, and females have increased risks for endometrial and ovarian cancer. Screening of Lynch syndrome is based on the Amsterdam Criteria II [33]: three or more relatives with an associated cancer (colorectal cancer or cancer of the endometrium, small intestine, ureter or renal pelvis), including one first-degree relative of the other two; two or more successive generations affected; one or more relatives diagnosed before the age of 50 years old; and exclusion of familial adenomatous polyposis (FAP) in cases of colorectal carcinoma. Definitive diagnosis is based on germline mutation analysis. Mutations of MMR genes, *MLH1*, *mutS homolog 2* (*MSH2*), *mutS homolog 3* (*MSH3*), *MSH6*, *postmeiotic segregation increased1* (*PMS1*) and *PMS2*, are involved in the pathological mechanism of Lynch syndrome [32,34] through inhibition of MMR during DNA replication, which leads to subsequent MSI and carcinogenesis [34].

Banno *et al*. [35] found a rate of 0.5% (2/375 patients) for cases of Lynch syndrome diagnosed based on the Amsterdam Criteria II among patients with endometrial cancer, which is extremely small compared with the 5% rate for colorectal cancer. In a multicenter study (five institutions) of 120 patients with endometrial cancer who met criteria A (one or more first-degree relatives with a Lynch syndrome-related cancer: colorectal, endometrial, small intestinal, ureteral or renal pelvis, gastric, ovarian or breast cancer; and one or more cancer diagnosed before the age of 50 years old) or criteria B (patients with two or more Lynch syndrome-related cancers), Hirai *et al*. [36] found 18 patients with MMR gene abnormalities and nine with *MSH6* mutations. Of 72 patients who met criteria A, 14 (19.4%) had MMR gene abnormalities, but none were diagnosed with Lynch syndrome by the Amsterdam Criteria II. These results show that *MSH6* is the most important MMR gene in endometrial cancer that may be related to Lynch syndrome and that the Amsterdam Criteria II identify only about half of the cases with germ cell mutation of MMR genes. Thus, these criteria do not allow definitive diagnosis of Lynch syndrome, but are useful for selecting patients who should undergo genetic tests. It is also important to pay attention to the presence or absence of mutations of MMR genes and, particularly, *MSH6*. In double cancers with simultaneous endometrial plus other HNPCC tumor types, the endometrial tumor seems to develop later, which is of interest from the perspective of screening for Lynch syndrome-related endometrial cancer [34]. Thus, examination of MMR genes and a detailed survey of family history in patients with endometrial cancer are important for the development of an appropriate treatment.

DNA methylation is also of interest in Lynch syndrome. Genomic imprinting is controlled by imprinting control centers (ICs) included in imprinting genes, and ICs control imprinting if just one allele is methylated. Therefore, DNA methylation plays an important role in imprinting, and these abnormalities may be a cause of genetic disease [37]. Gazzoli *et al*. detected aberrant *MLH1* methylation of one allele in DNA isolated from peripheral blood of one of 14 patients with suspected Lynch syndrome, but no mutation of *MSH2*, *MSH6* and *MLH1* in germ cell lines [38]. This patient developed Lynch syndrome, due to loss of heterozygosity in the non-methylated *MLH1* allele, with consequent inactivation of *MLH*. Thus, Lynch syndrome can occur without mutation of MMR genes, but with epimutation in the *MLH1* and *MSH2* promoter regions. Conversely, epimutation in germ cell lines may be a cause of Lynch syndrome, based on a family with mutation in the epithelial cell adhesion molecule (EPCAM) germ cell line, which causes hypermethylation in CpG islands in the *MSH2* promoter [39]. This epigenetic abnormality is also transmitted genetically.

Cancer associated with epimutation has specific histological characteristics based on unknown mechanisms. The methylation pattern in normal tissues may be a diagnostic indicator for the cancer tissue type [37] and can be determined based on tests using less invasive methods than conventional approaches. Comparison of methylation patterns of cancer patients and healthy individuals may ultimately permit prediction of the risk of cancer in healthy persons.

Cowden syndrome (CS) and Peutz-Jeghers syndrome (PJS) are also genetic diseases associated with endometrial cancer. CS is a rare disease with autosomal dominant inheritance and has characteristics of multiple hamartomas that occur in various tissues. The prevalence of CS is estimated to be one per 200,000–250,000 population [40]. Patients with CS have increased risks for malignant tumors and, particularly, breast, thyroid and endometrial cancers. The onset of CS involves the *PTEN* gene [41], and approximately 80% of patients with CS have a *PTEN* mutation [42]. The lifetime risk of endometrial cancer is 2% to 4% in the general population, but 5% to 10% in patients with CS [43]. *PTEN* codes for a protein with tyrosine kinase activity and acts as a tumor suppressor gene. PTEN is a phosphatidylinositol phosphatase that regulates the reverse reaction of PI3K and inhibits Akt activation via PI3K. Akt is a serine/threonine kinase that activates or inactivates downstream factors by phosphorylating serine/threonine residues, with resulting transmission of signals related to cell growth, survival, differentiation and glucose metabolism [44]. Akt is related to cell survival through inactivation of apoptosis executioners and transcription factors related to apoptosis-inducing factors. Therefore, if PTEN regulation of Akt activation is lost, the PI3K-Akt pathway is activated and leads to malignant transformation of cells.

Peutz-Jeghers syndrome (PJS) is characterized by multiple hamartomatous polyps in the gastrointestinal tract and mucocutaneous pigmentation. Patients with PJS have a higher risk of developing a malignant tumor in the gastrointestinal tract and other organs compared to the general population. *LKB1/STK11* has been identified as a disease-related gene with autosomal dominant inheritance, and *LKB1* mutation is found in 80% to 94% of patients with PJS [45]. The incidence of PJS is estimated to be one per 50,000–250,000 population [46]. Patients with PJS are at risk for gynecological cancers and have a 9% lifetime risk of developing endometrial cancer [47]. *LKB1* codes for a serine/threonine kinase that directly phosphorylates AMPK and has functions, including regulation of glucose, lipid metabolism, cell proliferation and cell polarity.

## 6. Biomarkers in Endometrial Cancer

The most important factors for diagnosis and outcome prediction in endometrial cancer are surgical stage, tumor differentiation, invasion depth and vascular invasion, based on the International Federation of Gynecology and Obstetrics (FIGO) guidelines. Useful histological biomarkers for endometrial cancer include *p53*, *PTEN*, MSI, *β-catenin*, Ras-mitogen-activated protein kinase (MAPK), extracellular signal-regulated kinase (ERK), vascular endothelial growth factor (VEGF) and DNA aneuploidy. Serum markers include carbohydrate antigen 125 (CA125), carbohydrate antigen 15-3 (CA15-3), chitinase 3-like 1 protein (YKL-40), VEGF and human epididymal secretory protein E4 (HE-4) (Table 3) [8,48].

Lee *et al*. [49] found that women with endometrial cancer associated with a *p53* mutation had an approximately 11-fold higher risk of death, due to endometrial cancer, compared to women without this mutation (95% CI: 1.01–120.7). This result is linked to the frequency of *p53* mutation in type 2 endometrial cancer. In contrast, *PTEN* is related to endometrioid adenocarcinoma with good prognosis and is often detected in Stage 1A tumors. Cases with *PTEN* mutations have a low recurrence rate [50], and Mackey *et al*. [51] found that patients with *PTEN* mutations had better outcomes (risk ratio: 0.55, 95% CI: 0.32–0.94) compared to those without *PTEN* mutations. Saegusa *et al*. [52] found a relationship of *β-catenin* mutations with histological low grade cancer and lymph node metastasis in 199 patients with endometrial cancer.

*K-ras* in the Ras-MAPK-ERK signaling pathway has been associated with patients with poor outcomes. Thus, Mizuuchi *et al*. [53] found a *K-ras* mutation in six of 49 patients, and three of those with mutations died during the follow-up period, compared to a death rate during follow-up of only 7% in the 43 patients without a *K-ras* mutation. *Ras-association domain family 1* (*RASSF1A*) is a tumor suppressor gene that regulates the Ras-MAPK-ERK signaling pathway. Jo *et al*. [54] found frequent *RASSF1A* methylation in cases with FIGO stage III and IV, lymph invasion and poorly-differentiated cancer and a five-year survival rate of 97.0% (*p* = 0.039) in patients with no *RASSF1A* methylation compared with 77.8% in those with *RASSF1A* methylation. For ERK, Mizumoto *et al*. [55] found significantly lower event-free survival in patients with low expression of *phosphorylated ERK* (*pERK*) (*p* = 0.047).

VEGF is an endothelial mitogenic factor, and increased VEGF expression is associated with highly malignant endometrial cancer, deep muscle layer invasion, vascular invasion and lymph node metastasis. Chen *et al*. [56] showed that a high VEGF concentration in the cytoplasm is often observed in Stage II or higher cancer, and that a level >800 pg/mg is a risk factor for recurrence, similarly to a poorly-differentiated (G3) cancer.

Cases involving DNA aneuploidy account for 16% to 28% of all endometrial cancer and typically involve elderly patients with non-endometrioid adenocarcinoma, high malignancy and lymph node metastasis. Zaino *et al*. [57] showed that patients with cancer associated with DNA aneuploidy had a 4.1-fold higher risk (95% CI: 2.3–7.3) for death, due to cancer, compared with patients with diploidy.

CA125 is currently recognized to be a marker for recurrence in the guidelines for endometrial cancer in Japan. The serum marker CA125 is increased to >35 U/mL in 11%–34% of patients with endometrial cancer and is associated with stage, muscular invasion, cervical invasion, peritoneal cavity biopsy and lymph node status. In a study of 148 patients, Scambia *et al*. [58] found a CA125 level ≥65 U/mL in just 22% of lymph node metastasis-negative patients, but in 58% of lymph node metastasis-positive patients (*p* = 0.0220). Sood *et al*. [59] found that patients with CA125 > 65 IU/mL had metastatic lesions at a rate 6.5-fold higher (95% CI: 2.5–17.1) than that in patients with CA125 < 65 IU/mL. High preoperative CA125 is also common in patients with poor outcomes, with Scambia *et al*. [58] finding a significant correlation between CA125 > 65 IU/mL and a short survival period (*p* = 0.0027). Serum antibody CA15-3 is also increased in patients with endometrial cancer and may be related to an advanced cancer stage. Thus, CA15-3 > 30 U/mL was found in 47% of patients with Stage III endometrial cancer, but only in 18% of Stage I or II cases (*p* = 0.01) [58], and a CA15-3-positive status (>30 and >50 U/mL) had significant correlations with a short survival period (*p* = 0.0004 and *p* = 0.00025). Increased levels of glycoprotein YKL-40, a member of the chitinase family, also suggests a poor outcome, with Diefenbach [60] finding a significantly lower five-year survival rate of 43% in patients with YKL-40 > 80 ng/mL compared to the rate of 80% in other patients (*p* = 0.004). Finally, for HE-4, an investigation of serum specimens of 156 healthy women and 171 patients with endometrial cancer by Moore *et al*. [61] showed that HE-4 had 12.9% (ROC AUC) higher accuracy as a marker for early endometrial cancer in combination with CA125, compared to CA125 alone (*p* < 0.0001). Mutz-Dehbalaie *et al*. [62] used multivariate analysis to show that serum HE4 before intervention is an independent prognostic predictor of overall survival. The hazard ratio for HE4 > 81 pmol/L compared to <81 pmol/L was 2.407 (*p* = 0.017). In combination with CA125, the hazard ratio for overall survival for HE4 > 81 pmol/L and CA125 > 35 U/mL compared to values below these cutoffs was 4.04 (*p* = 0.023), and CA125 was shown to be an independent prognostic factor in multivariate analysis. The sensitivity and negative predictive value for HE4 were 94% and 97%, respectively, in endometrial cancer at stages IA and IB. These results indicate that serum HE4 is useful for screening for endometrial cancer [62].

Collectively, these results suggest that *PTEN* and *β-catenin* mutations are markers of good outcomes in endometrial cancer, while *p53*, DNA aneuploidy and increased serum CA125 predict poor outcomes. Positive use of these markers with conventional prognostic factors may permit early definition of risks for individual patients and appropriate adjustment of treatment procedures.

## 7. Conclusions

Improvements in histology and diagnostic imaging have simplified diagnosis of endometrial cancer, but it remains difficult to define individual risks accurately, and treatment is still chosen based on the conventional FIGO stage. However, identification of epigenetic mutations, miRNA abnormalities and other biomarkers has the potential for early diagnosis and prediction of risks and outcomes using specimens obtained by low-invasive methods, such as blood drawing. Examinations and screening using these techniques will allow definition of accurate and detailed pathology and the choice of optimal treatment options in personalized medicine.

## Figures and Tables

**Table 1:** Genetic mutations in Type 1 and 2 endometrial cancer.

Genetic alteration	Type 1 (%)	Type 2 (%)
*PTEN* inactivation	50–80	10
*K-ras* mutation	15–30	0–5
*β-catenin* mutation	20–40	0–3
Microsatellite instability	20–40	0–5
*p53* mutation	10–20	80–90
*HER-2/neu*	10–30	40–80
*p16* inactivation	10	40
*E-cadherin*	10–20	60–90

**Table 2:** Expression of miRNAs in endometrial cancer.

Upregulation	Downregulation
miR-200a	miR-410
miR-200b	miR-15b
miR-200c	miR-17-5p
miR-429	miR-20a
miR-203	miR-34b
miR-205	miR-152
miR-210	miR-125a
	miR-214
	miR-221
	miR-222
	miR-424

**Table 3:** Biomarkers in endometrial cancer.

Tissue biomarkers	Serum biomarkers
*p53*	CA125
PTEN-PIK3-mTOR signaling pathway (*PTEN*, *PIK3*, *mTOR*, *4E-BP1*)	CA15-3
MSI	YKL-40
*β-catenin*	VEGF
Ras-MAPK-ERK signaling pathway (*K-ras*, *RASSF1A*, *ERK*)	HE-4
VEGF	
DNA aneuploidy	
